# Real-Time Monitoring of the Cytotoxic Effect of Oxygen-Sensitive Fluorescent Poly(styrene-maleic anhydride) Nanoparticles Using Electrical-Substrate Impedance Sensing

**DOI:** 10.1021/acsabm.5c01443

**Published:** 2025-10-09

**Authors:** Fernando Pesantez Torres, Elijah C. Feret, Yubing Xie, Susan T. Sharfstein

**Affiliations:** † Department of Nanoscale Science and Engineering, College of Nanotechnology, Science and Engineering, University at Albany, State University of New York, 257 Fuller Road, Albany, New York 12203, United States; ‡ The RNA Institute, University at Albany, State University of New York, 1400 Washington Avenue, Albany, New York 12222, United States

**Keywords:** nanoparticles, oxygen sensing, electrical cell−substrate impedance sensing (ECIS), cytotoxicity, barrier function

## Abstract

Fluorescent poly­(styrene-*co*-maleic anhydride) (SMA) nanoparticle (NP) oxygen sensors show strong potential for visualizing in situ oxygen gradients in biomedical research. To expand their applications, it is essential to understand their cellular interactions. Electrical cell–substrate impedance sensing (ECIS) enables real-time monitoring of cell behavior by measuring the impedance of monolayer cultures with an alternating current. In this study, we used ECIS to assess the cytotoxicity of SMA NP oxygen sensors and further examined their effects through endothelial barrier function analysis and microscopy. The sensors showed no cytotoxicity at any dose, confirming their biocompatibility. However, the NPs incorporated into the extracellular matrix and may disrupt barrier function. These findings support further use of SMA NP oxygen sensors in biomedical research and highlight ECIS as a valuable tool for evaluating nanoparticle biocompatibility when traditional optical assays are limited by optical interference.

## Graphical abstract

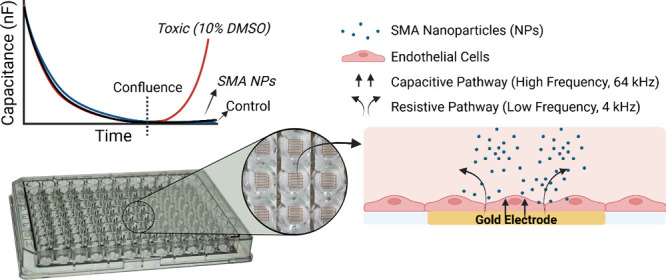

## 1 Introduction

Nanoparticles (NPs) have shown unique applications in drug delivery, imaging, and diagnostics, and theranostics.^1^ In particular, fluorescent poly­(styrene-*co*-maleic anhydride) (SMA) NPs are able to encapsulate and transport hydrophobic dyes. Among these dyes, PtTFPP (Pt­(II)) (*meso*-tetra­(pentafluorophenyl)­porphine) is particularly useful for oxygen sensing as it exhibits oxygen quenching in a repeatable and predictable manner.^2^ PtTFPP encapsulated in NPs has been used as a sensor for biological applications, ranging from in vivo imaging studies of oxygen distributions throughout multicellular organisms to targeted, subcellular oxygen dynamics in cancers.^3–5^

However, as the use of SMA NP sensors expands, so does the need to rigorously evaluate their safety when interacting with living systems. Traditional cytotoxicity assays used to assess cell viability include MTT ((4,5-dimethyl-2-thiazolyl)-2,5-diphenyl-2*H*-tetrazolium bromide or methyl thiazolyl tetrazolium), alamarBlue, or LIVE/DEAD assays.^3,4^ However, these assays present certain disadvantages. MTT is often used as a cytotoxicity assay, and while effective, it has multiple pitfalls. It is highly susceptible to optical interference, which can render it ineffective if there are other fluorophores or particles that can scatter or absorb light.^6^ Further, MTT is an end point assay and requires killing cells to extract data, limiting data collection to a single time point. AlamarBlue or resazurin addresses the limitation of MTT as an end point assay, as alamarBlue can be used to assess viability, then be removed, and the cell culture can continue, although it is still an optical assay that is vulnerable to optical interference.^7^ LIVE/DEAD staining provides an effective measure of spatial distribution of viable and dead cells, but it does not provide real-time cytotoxicity measurements of the NP sensors. LIVE/DEAD staining has been used to measure the cytotoxic effect of SMA NP sensors within a bioprinted construct, in situ.^3^ However, these cells and NPs were not freely interacting; they were both mixed with the bio-ink, and therefore, were partially secured in place.

In contrast, electrical-substrate impedance sensing (ECIS) is a real-time monitoring technique that offers the advantage of continuously tracking cellular behavior, thereby capturing transient changes in cell morphology, adhesion, and viability.^8,9^ This capability becomes important when assessing the cytotoxic effects of nanomaterials, where slight alterations can have implications for both therapeutic efficacy and safety. ECIS is a powerful platform for label-free, real-time cytotoxicity monitoring, offering a distinct advantage over traditional assays.^10–12^ In addition to these advantages, ECIS provides the unique benefit of high-throughput analysis of cell-material interactions and cell behaviors (e.g., cell adhesion, proliferation, migration, invasion) with exceptional sensitivity,^13–16^ enabling measurement of the cell–substrate distance at the nanoscale and detection of subtle cellular changes beyond cell death even in single cells.

Here, we applied ECIS to monitor the real-time cytotoxic effect of fluorescent SMA NP-based oxygen sensors on multiple adherent mammalian cell cultures. Our approach aims to eliminate the potential for optical interference by using electrical impedance to measure cellular confluence, instead of relying on fluorescent intensity to measure cellular metabolism. This method overcomes the limitations of conventional end point cytotoxicity assays, allowing for real-time monitoring of the interaction between cells and NPs without any additional assay reagents. In this study, we first synthesized fluorescent SMA NPs using emulsifier-free nanoprecipitation. We subsequently characterized these NPs using scanning electron microscopy (SEM), dynamic light scattering (DLS), and UV–vis spectroscopy/spectrofluorimetry. We then measured the cytotoxicity of SMA NPs on two model cell lines, NIH 3T3 fibroblasts and bEnd3 brain epithelial cells, using the alamarBlue assay and tested the feasibility of utilizing the ECIS system to assess the cytotoxic effect of these NPs. ECIS provides a new avenue for noninvasive, real-time monitoring of polymeric NP biosensors, as well as other potential fluorescent toxicants that may interfere with traditional cytotoxicity assays.

## 2 Materials and Methods

### 2.1 Synthesis of Fluorescent SMA NPs

SMA NPs were synthesized based on a previously described protocol using an emulsifier-free nanoprecipitation.^5^ Briefly, 200 mg of Xibond 120 SMA (Aurorium, Indianapolis, IN), 3 mg of Macrolex Fluorescent Yellow 10GN (MY; sold as 3-(5-chloro-1,3-benzoxazol-2-yl)-7- (diethylamino)-2*H*-chromen-2-one by Sigma-Aldrich; St. Louis, MO), and 3 mg of PtTFPP (Frontier Specialty Chemicals, Logan, UT) were dissolved in 22.5 mL of tetrahydrofuran (THF; Sigma-Aldrich) and mixed thoroughly. This mixture was then poured into 200 mL of heavily stirred deionized water (DI water) and stirred for 20 min to allow the THF to evaporate. The initial NP concentration was quantified by drying and weighing 1 mL of suspension. NP suspensions were stored in the dark at room temperature and were vortexed to ensure full resuspension prior to use.

### 2.2 Characterization of Fluorescent SMA NPs

#### 2.2.1 UV–Vis Spectroscopy/Spectrofluorimetry

UV–vis spectroscopy/spectrofluorimetry were performed to measure the absorbance, excitation, and emission spectra of these fluorescent SMA NPs. For Beer–Lambert’s law calculation, the optical path length of the vessel (i.e., 96-well plate) and absorbance values of NP samples of known concentration were determined. The path length of the 96-well plate used was calculated using water. 100 μL of DI water was added to multiple wells, and the absorbance of the water was measured at 977 nm with 900 nm as a reference wavelength using a Tecan Infinite M200 Pro microplate reader (Tecan Group Ltd., Männedorf, Switzerland). The difference in absorbance at 977 and 900 nm was calculated and divided by the known molar absorption coefficient of water (0.178 mol^–1^ cm^–1^). The numerical average (*n* = 21) was used as the path length.

A serial dilution of SMA NPs with a known concentration was completed at a ratio of 1:2 (NP suspension of known concentration:DI water). The known concentration of the initial solution was found by measuring the dry weight of 1 mL of the suspension. The serial dilution was repeated 10 times to give 11 known concentration groups as well as a DI water control group. The absorbance was measured at 425 nm, and background absorbance detected in water was subtracted. The numerical average (*n* = 5) of absorbance at each concentration was used to create a standard curve (absorbance vs NP concentration). Based on the standard curve, the concentration of the NP dilution being added to the cell culture was determined by measuring the absorbance as above and applying Beer–Lambert’s Law. NP concentrations were measured prior to addition to cell culture medium, and final concentrations were calculated based on the dilution.

Fluorescence excitation and emission spectra were also measured using the Tecan Infinite M200 Pro microplate reader (Tecan Group Ltd., Männedorf, Switzerland). Emission spectra were measured by exciting the sample at 425 nm and measuring emission from 450 to 700 nm. Excitation spectra were obtained by varying the excitation wavelength from 350 to 500 nm while measuring the emission at 488 and 510 nm, the literature and observed emission maxima for Macrolex Yellow, respectively, as well as at 650 nm, the emission maximum of PtTFPP. In all cases, 100 μL of stock NP suspension (approximately 1 mg/mL) was added to each well of a 96-well plate. For low oxygen measurements, nitrogen gas was gently bubbled into the well for 25 min and measurements were taken promptly thereafter.

#### 2.2.2 Dynamic Light Scattering (DLS)

NP size and size distribution were measured using dynamic light scattering (DLS) using a Malvern Zetasizer Nano ZS (Malvern Instruments, Malvern, U.K.) with a 633 nm He–Ne laser. NP suspensions were diluted in DI water to 0.1% of stock concentration and were filtered through a 0.45 μm syringe filter to remove any large aggregates. The concentration was then assessed by measuring the absorbance in a 96-well plate using the previously acquired specific absorption coefficient. DLS measurements were completed at 25 °C at a fixed measurement angle of 173°. The resulting intensity autocorrelation function was analyzed using the cumulant method to find the hydrodynamic diameter and polydispersity index (PDI). The reported size distribution, average size, and polydispersity index reported are the numerical averages of five samples measured.

#### 2.2.3 Scanning Electron Microscopy (SEM) of NPs

Droplets of NP suspension were placed on a fragment of silicon wafer and allowed to air-dry at room temperature overnight. Samples were sputter-coated with iridium, placed on the specimen holder, and imaged with the Zeiss LEO 1550 FE-SEM microscope (Zeiss Leo Electron Microscopy Ltd., Cambridge, U.K.). Image analysis was completed using the open-source image analysis software ImageJ (https://imagej.net/).

### 2.3 Cell Culture

#### 2.3.1 bEnd3 Brain Endothelial Cells

bEnd3 murine brain endothelial cells, obtained from the American Type Culture Collection (ATCC, CRL-2299, VA), were cultured in Gibco DMEM (low glucose) supplemented with 10% fetal bovine serum (Sigma-Aldrich), 0.1% gentamicin (Thermo Fisher Scientific). Cells were incubated in a 37 °C, 5% CO_2_ humidified incubator and subcultured every 3 days when they were 80–90% confluent.

#### 2.3.2 NIH 3T3 Fibroblasts

Murine NIH 3T3 fibroblasts^19^ were cultured in Gibco DMEM (high glucose 4.5 g/L) supplemented with 10% fetal bovine serum, 1% penicillin-streptomycin (10,000 units/mL of penicillin and 10,000 μg/mL of streptomycin) (Thermo Fisher Scientific). Cells were incubated in a 37 °C, 5% CO_2_ humidified incubator and subcultured every 3 days when they were 80–90% confluent.

### 2.4 Cell Analysis

#### 2.4.1 ECIS Real-Time Measurements

Before cell inoculation, each well of a 96-well plate (96W1E+ PET ECIS and 96W20idf PET ECIS, Applied Biophysics, Troy, NY) was treated with 100 μL of 10 mM cysteine solution (Applied Biophysics) in water for 15 min to stabilize the electrodes. The cysteine solution was removed, and 30000 cells in 200 μL of cell culture medium were immediately added to each well. Finally, the 96-well plate was connected to the ECIS 96-well station (Applied Biophysics), and measurements were performed at four different frequencies, 400, 4000, 32000, and 64000 Hz, simultaneously collecting impedance, resistance, and capacitance values. Cells were cultured for 48 h in a humidified incubator at 37 °C in 5% CO_2_. The medium was removed and fresh media with five concentrations (146, 91, 50, 35, and 25 μg/mL) of fluorescent SMA NPs were added to the respective wells, and real-time measurements were taken using an ECIS ZTheta instrument (Applied Biophysics) for 3 days. Wells with cells in 10% (v/v) DMSO were used as a cytotoxicity positive control. For barrier analysis, a 96W20idf PET ECIS well plate (Applied Biophysics) was used, and SMA NPs were added after 4 days of culture. All subsequent analyses were performed using cells cultured in these 96-well plates.

The lowest NP dose that reliably produced reference fluorescence under microscopy (25 μg/mL) was selected as the minimum dose in this study, as a reference signal is required for all applications. The maximum dose of 146 μg/mL was chosen to allow for cell–NP interaction while remaining within the practical range for freely suspended NPs in cell culture. This dose represents a balance between doses previously reported. In a study of intracellular oxygen concentrations, a 20 μg/mL suspension of poly-l-lysine-coated oxygen-sensing SMA NPs and polystyrene NPs was added to cell culture, but in that case, poly-l-lysine promoted cell-NP interactions.^20^ In another study, oxygen-sensitive SMA NPs were added to a dense hydrogel bio-ink at a concentration of 2.5 mg/mL; however, the hydrogel acted to limit the cell–NP interactions.^3^

#### 2.4.2 Fluorescence and Phase Contrast Microscopy

Fluorescence microscopy was performed using the EVOS M7000 Imaging System (Thermo Fisher Scientific) to visualize fluorescent SMA NPs in cell culture directly from the 96-well plate, utilizing the EVOS GFP 2.0 light cube (ex, 470/22 nm; em, 525/50 nm). Phase contrast microscopy was performed concurrently on the same samples.

#### 2.4.3 alamarBlue Assay

Viable cell growth and cytotoxicity of NP-treated NIH 3T3 fibroblasts and bEnd3 brain endothelial cells were evaluated by alamarBlue assay in which viable cells reduce resazurin into a highly fluorescent resorufin. Cells without any NP contact were used as the untreated control and cells treated with 10% DMSO were used as the cytotoxicity positive control. At the conclusion of NP contact, 200 μL of medium containing 10% (v/v) Invitrogen alamarBlue reagent (Thermo Fisher Scientific) was added to each well, followed by incubation for 2 h at 37 °C and 5% CO_2_. Medium samples were subsequently removed from each well and placed in a 96-well plate and analyzed using a TECAN Infinite M200 microplate reader. Fluorescence intensity was measured at an excitation wavelength of 545 nm and emission wavelength of 590 nm. To calculate the cytotoxicity percent of each of the SMA NPs concentrations, the following formula was used:

1 $$\mathrm{cytotoxicity}\left(\%\right)=\frac{\mathrm{sample}-\mathrm{negative control}}{\mathrm{positive control}-\mathrm{negative control}}\times 100$$

where the negative control represents only cells, and cells treated with 10% (v/v) DMSO were used as a positive control.

#### 2.4.4 Scanning Electron Microscopy (SEM)

SEM analysis was performed to evaluate cell morphology and visualize the interaction of the cells with the NPs. Cells grown in ECIS well plates (as described above) were fixed and imaged as previously described.^21^. Briefly, cells were fixed with 3% glutaraldehyde (Sigma-Aldrich) in 0.1 M phosphate buffer (pH 7.4) (Sigma-Aldrich) containing 0.1 M sucrose (Sigma-Aldrich) at room temperature for 2 h. After fixation, samples were washed 3 times with 0.1 M phosphate buffer and dehydrated in a series of graded ethanol incubations (Thermo Fisher Scientific) for 15 min at each concentration. Samples were then dried with hexamethyldisilane (Sigma-Aldrich). Finally, the bottom of the 96-well plate containing the electrodes and cell culture samples was removed to allow SEM imaging. The samples were sputter-coated with iridium, placed on the specimen holder, and imaged with the Zeiss LEO 1550 FE-SEM microscope (Zeiss Leo Electron Microscopy Ltd., Cambridge, U.K.). An accelerating voltage of 2 kV was used for both cell types.

### 2.5 Statistical Analysis

Data are represented as mean ± standard deviation. Experiments were repeated at least three times. Statistical analysis was performed using one-way and two-way ANOVA comparing the different samples to the control using GraphPad Prism 10.4.1. software. A *p*-value < 0.01 was considered statistically significant.

## 3 Results and Discussion

### 3.1 Physical and Optical Characterization of Fluorescent SMA NPs

Oxygen level is an important factor when culturing cells, whether in a monolayer, suspension, or three-dimensional microenvironments. Oxygen-sensitive, fluorescent SMA NPs have significant potential for in vitro oxygen monitoring in engineered tissue constructs.^3^ To begin our investigation into the interaction between cells and oxygen-sensing fluorescent SMA NPs, we first synthesized the NPs using an emulsifier-free nanoprecipitation. To corroborate the successful synthesis of the fluorescent SMA NPs, we characterized NP morphology, size distribution, and optical properties using SEM, DLS, and UV–vis spectroscopy/spectrofluorimetry.

#### 3.1.1 SMA NP Sizing

SEM showed that these SMA NPs were spherical in shape (Figure 1a), and further analysis using ImageJ yielded an average NP diameter of 166.7 ± 58.0 nm (*n* = 233). To evaluate the hydrodynamic size of these synthesized fluorescent SMA NPs, DLS was completed using a 0.1% dilution of the NP suspension in DI water, passed through a 0.45 μm syringe filter to remove large aggregates. Following the filtration, the solution concentration was measured using the previously described Beer’s law method and was found to be approximately 6 μg/mL. DLS revealed an average hydrodynamic diameter of 216.0 ± 16.0 nm and a PDI of 0.15 ± 0.03; using the average PDI, an average size standard deviation of 81.6 ± 8.8 nm was found. These values are comparable to previous literature values for fluorescent SMA NPs synthesized using emulsifier-free nanoprecipitation.^3,5,17^ The particle size distribution given by DLS is shown in Figure 1b. The differences between these size measurements could be attributed to sample variation, varying measurement parameters, and deswelling during drying. DLS measures the hydrodynamic diameter of NPs in solution, whereas SEM measures the physical diameter. SMA is known to swell in water and thus, deswell when dried, as was done for SEM imaging.^22^ This difference has been previously described with similar SMA NPs.^17^

**Figure 1:**
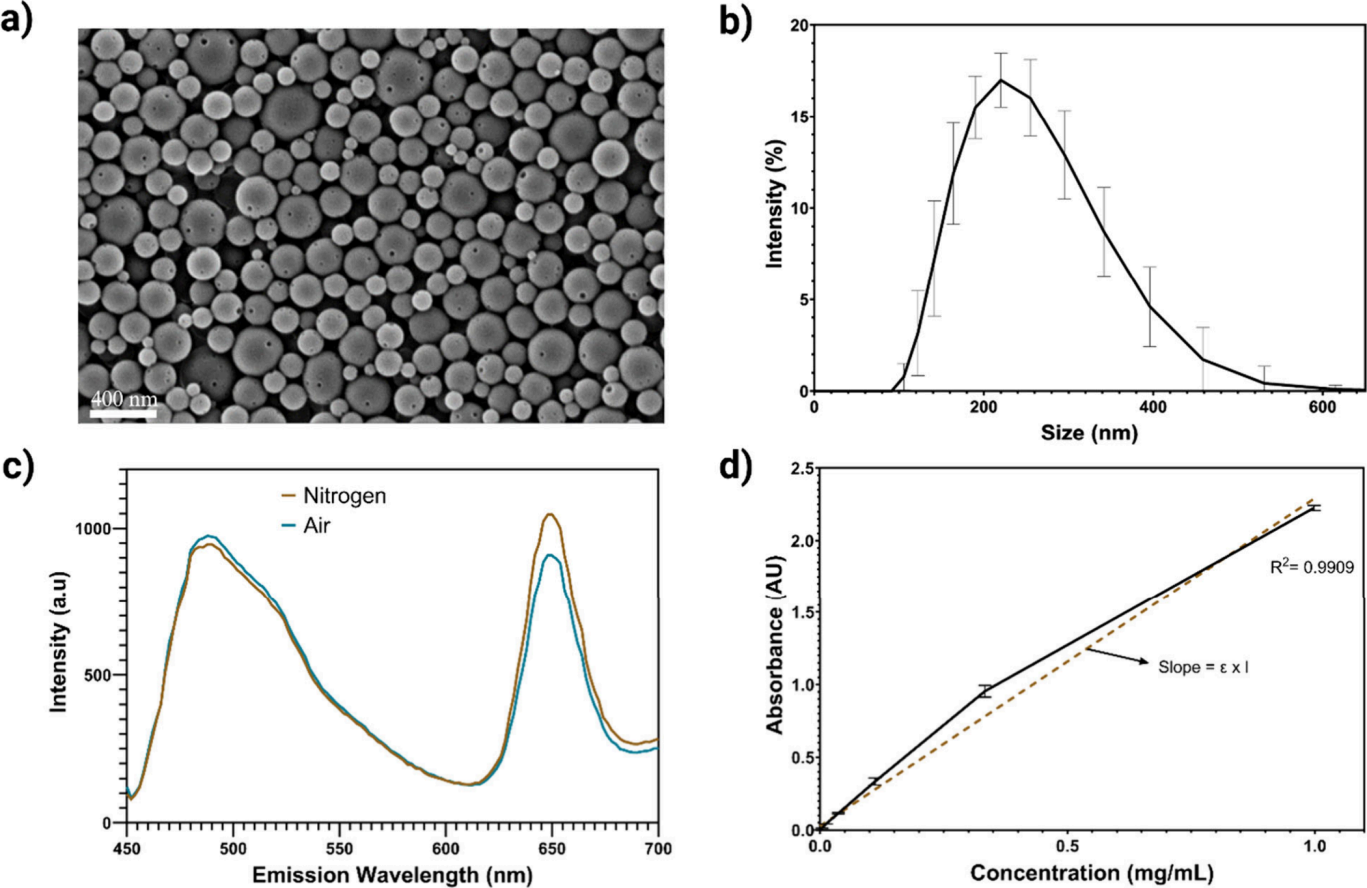
Characterization of SMA NPs. (a) SEM micrograph of SMA NPs. Scale bar = 400 nm. (b) Dynamic light scattering measurement showing the size distribution of SMA NPs. (c) Emission spectra of SMA NPs at 425 nm excitation wavelength. (d) Beer–Lambert Law standard curve of absorbance (at 425 nm) vs concentration of SMA NPs.

#### 3.1.2 UV–Vis Spectroscopy/Spectrofluorimetry

UV–vis spectrofluorimetry was used to investigate the excitation and emission wavelengths of the NPs as well as to obtain a calibration plot. Fluorescence spectra were obtained by adding 100 μL of stock concentration NP suspension (approximately 1 mg/mL) to a 96-well plate. The suspensions were then either allowed to acclimatize in the air or were bubbled with nitrogen to remove most of the dissolved oxygen, a previously described approach.^23^ The emission spectrum (Figure 1c) with excitation at 425 nm showed a strong peak at 488 nm across aerobic and anaerobic samples and a variable intensity peak at 650 nm, which was higher in the samples exposed to nitrogen. The peak observed at 488 nm can be attributed to the MY, which emits in the green domain, although the known emission maximum is 512 nm.^24^ This blue shift is likely due to encapsulation of the dye within a polymeric NP suspended in water. This discrepancy could also be due to microenvironmental differences compared with prior studies, which employed dye encapsulation in poly­(methyl methacrylate) rather than SMA. The peak observed at 650 nm is attributed to the PtTFPP, which has been well characterized by its oxygen-quenchable peak at that wavelength. The emission spectrum that we observed had a peak in the red region and resembled the prior emission spectra for PtTFPP in air and nitrogen.^2^

The excitation spectra (Supporting Information Figures S1 and S2) were measured in a similar fashion to the emission spectra. Excitation wavelengths were varied from 350 to 500 nm and emission was measured at the literature-reported emission maxima for MY and PtTFPP, as well as the observed maximum for MY. In the green regime, regardless of the measured emission wavelength, there was a broad peak between 415 and 450 nm excitation wavelength. When measuring emission at 488 nm, spillover began at 460 nm. The shape and intensity of the values were otherwise nearly identical. When measuring emission at 650 nm, the shapes of the excitation spectra were the same for both the aerobic and anaerobic samples, although the sample purged with nitrogen showed consistently higher emission-intensity values. The excitation range is broad with local maxima at 390 and 450 nm. An excitation wavelength of 425 nm was selected for excitation of both MY and PtTFPP within these NPs due to its ability to excite both dyes, without causing spillover at the observed MY emission maximum.

UV–vis spectroscopy was used to create a standard curve of the absorbance intensity as a function of SMA NP concentration. Standardization was performed by using a serial dilution of the NP suspension. Using the known concentration of the stock solution, we created a standard curve of NP concentration and absorbance (Figure 1d). According to Beer–Lambert Law:

A=εlC

where *A* is absorbance, *ε* is the specific absorption coefficient, *l* is the length of the light path, and *C* is the NPs suspension concentration. The slope of this curve is the path length multiplied by the molar absorptivity. By using the previously calculated path length of water, 0.313 cm, we were able to determine that the NP suspension has an extinction coefficient of 7.226 mL mg^–1^ cm^–1^, which was further used to calculate the concentration of NP suspensions using Beer–Lambert Law.

### 3.2 End Point Cell Viability and Cytotoxicity Assessment of NIH 3T3 Fibroblasts and bEnd3 Brain Endothelial Cells Using alamarBlue Assay

To assess the toxicological effects of the oxygen-sensitive, fluorescent SMA NPs, two-day-old cultures of NIH 3T3 fibroblasts and bEnd3 brain endothelial cells were exposed to SMA NPs at various concentrations for 3 days followed by alamarBlue assay on day five. The alamarBlue assay uses the nonfluorescent blue dye, resazurin, which is reduced to the highly fluorescent pink resorufin in metabolically active cells; high fluorescence represents more metabolism and is equated with a higher number of viable cells.

Panels a and d of Figure 2 show fluorescence intensity and the statistical difference (one-way ANOVA, *p* < 0.0001) compared to the untreated control for the NIH 3T3 fibroblast groups and the bEnd3 brain endothelial cell groups, respectively (Control; 146, 91, 50, 35, and 25 μg/mL; 10% (v/v) DMSO). For NIH 3T3 fibroblasts, all SMA NP groups showed statistically significantly lower fluorescence compared to the untreated control. According to ISO 10993-5:2009, a decrease in cell viability greater than 30% (70% cell viability) is an indicator of cytotoxic effect.^25^ As seen in the relative cell density plots (Figure 2b,e), all groups treated with SMA NPs had relative cell viabilities of at least 80%, which is well above the 70% threshold for cytocompatibility. In brain endothelial cells, no statistical difference was observed between the different SMA NP groups and the untreated control (Figure 2d,e). Some treated groups (91 and 25 μg/mL) showed greater fluorescence than the control, though within the margin of error. This result is also shown in Figure 2c,f, which indicate that all groups have a cytotoxicity of less than 20%, which further confirms the biocompatibility of these SMA NPs.

**Figure 2:**
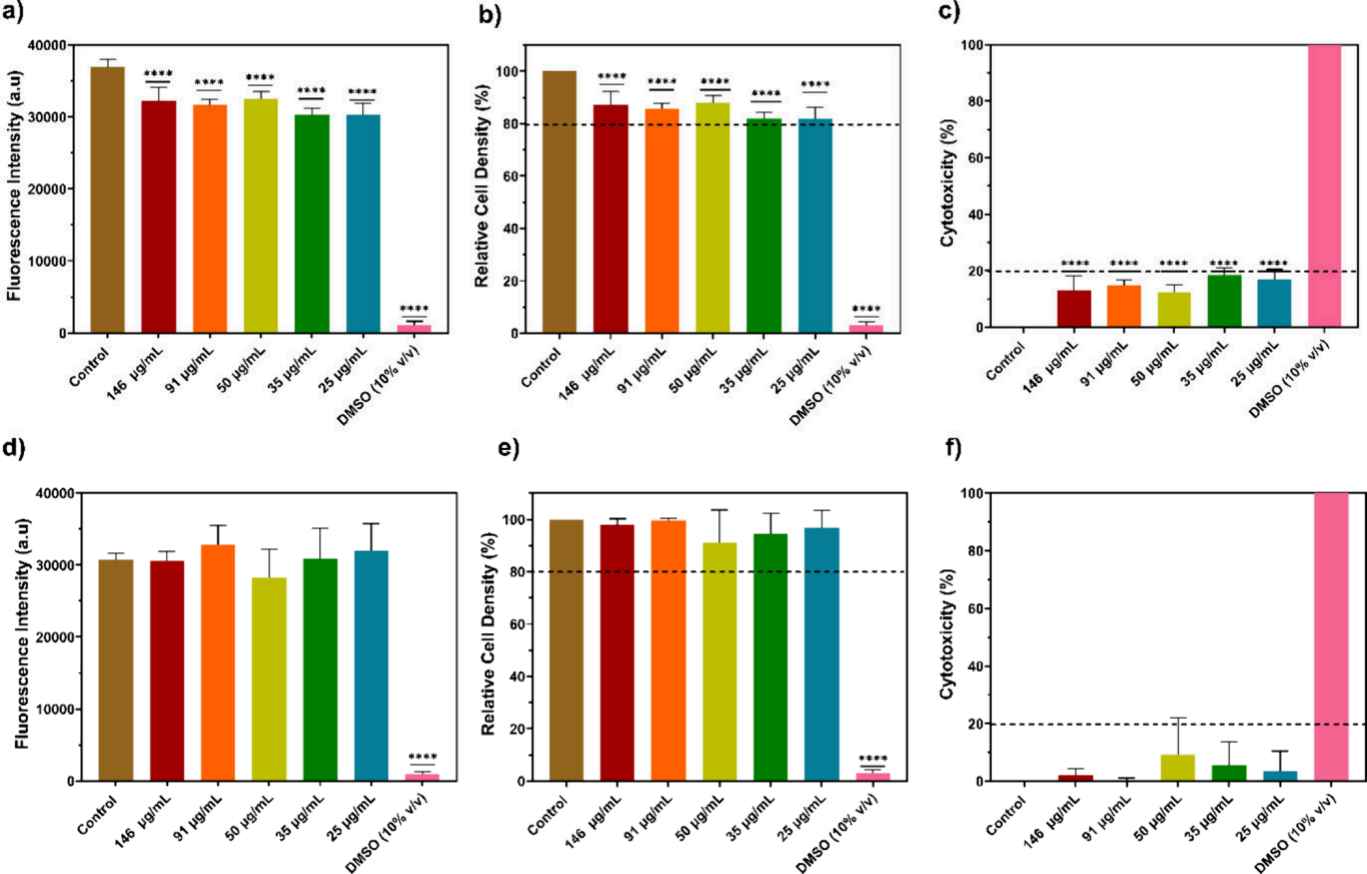
Effects of SMA NPs on viable cell density and toxicity of NIH 3T3 fibroblasts and bEnd3 brain endothelial cells assessed by alamarBlue assay: (a–c) NIH 3T3 fibroblasts; (d–f) bEnd3 cells. (a) Fluorescence intensity of reduced resazurin by NIH 3T3 fibroblasts on day 5 of culture corresponding to 3 days of incubation with SMA NPs. (b) Relative cell density of NIH 3T3 fibroblasts on day 5. (c) Cytotoxicity of SMA NPs on NIH 3T3 fibroblasts. (d) Fluorescence intensity of reduced resazurin by bEnd3 cells on day 5 of culture corresponding to 3 days of incubation with SMA NPs. (e) Relative cell density of bEnd3 cells on day 5. (f) Cytotoxicity of SMA NPs on bEnd3 cells. In all panels, the fluorescence intensity is compared to the negative control (brown bars). **** *p* < 0.0001. *n* = 4.

### 3.3 ECIS Real-Time Monitoring of Cytotoxic Effects of Fluorescent SMA NPs on NIH 3T3 Fibroblasts and bEnd3 Brain Endothelial Cells

In this study, we tested the feasibility of employing ECIS for cytotoxicity assessment by monitoring real-time changes in cell monolayer integrity in response to fluorescent SMA NPs. ECIS measures impedance (*Z*) changes that correlate with cellular behaviors, such as attachment, proliferation, and barrier function. This measurement is enabled by the application of a small alternating current (AC) to gold electrodes that serve as cell substrates. As cells attach and spread on the electrodes, the current is impeded, resulting in a potential across the cell layer. Impedance (*Z*) can be calculated by using Ohm’s Law, *Z* = *V*/*I*, where *I* is the current. Impedance measurements have been used previously to assess cytotoxicity of nanoplastics.^26^ However, here we employed complex impedance,

$$Z=\surd (R^{2}+{\left(X_{l}-X_{c}\right)}^{2}$$

where *R* is the pure resistance, *X*_l_ is the inductive reactance, and *X*_c_ is the capacitive reactance. This approach allows us not only to measure simple impedance, but also to measure resistance and capacitance separately, where capacitance (*C*) is calculated as $C=\frac{1}{2\pi fX_{c}}$ , where *f* is the measurement frequency in Hz. Some researchers have used complex impedance instead of simple impedance to study the cytotoxic effects of ZnO_2_ NPs^27^ and cannabidiol.^12^ In this work, we focused on capacitance values since, at higher frequencies (over 32000 Hz), current tends to follow a capacitive pathway through the cell membrane instead of flowing into the solution (medium) between adjacent cells. Therefore, capacitance values tend to offer a better insight into attached cells.

Figure 3a shows a real-time measurement of capacitance values of an NIH 3T3 fibroblastic monolayer for 5 days, while Figure 3d shows the same measurements for bEnd3 brain endothelial cells. After 2 days in culture, five different concentrations of SMA NPs (146, 91, 50, 35, and 25 μg/mL) were added to NIH 3T3 fibroblasts and bEnd3 brain endothelial cells and incubation continued for three additional days. An untreated control and a positive control (addition of 10% (v/v) DMSO) were also performed. Immediately after adding DMSO, cells began dying and detaching, resulting in increased capacitance values. However, wells with the NPs did not show any noticeable change in capacitance and were not significantly different from the untreated control after 3 days of incubation with NPs (Figure 3b,d). Capacitance values continued decreasing for all live groups due to extracellular matrix (ECM) formation. As performed for the alamarBlue assay, cytotoxicity percentage (Figure 3c,f) was calculated by assuming 0% for control wells and 100% for wells containing 10% (v/v) DMSO. All values were lower than 10% with no significant difference. Differences in capacitance curves between panels a and b of Figure 3 are due to different capacitance and resistance signatures between cell types, which could be attributed to various factors such as adhesion, size/geometry, membrane resistance, barrier formation, or cytoplasmic conductivity.^28,29^

**Figure 3:**
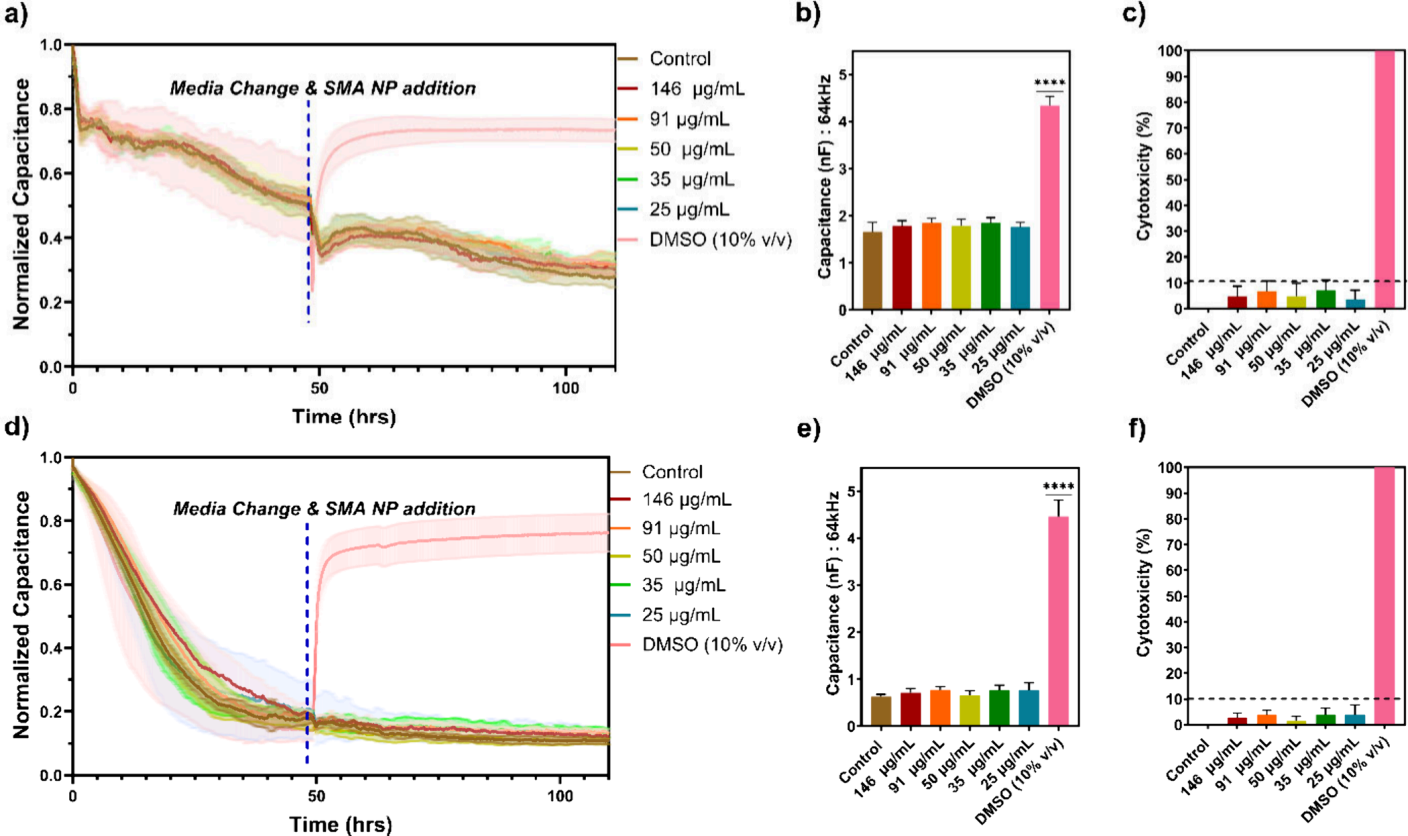
ECIS measurements of cellular response to the introduction of fluorescent SMA NPs. (a) Normalized five-day real-time ECIS measurement of NIH 3T3 fibroblasts, showing real-time data of cell attachment, proliferation, and response to fluorescent SMA NPs at varying concentrations. Capacitance was recorded at 64 kHz. The dotted blue line indicates the time when the fluorescent SMA NPs were added to each well. (b) Capacitance values from NIH 3T3 cells on day five at 110 h in culture. (c) Cytotoxicity percentage of NIH 3T3 fibroblasts in response to contact with SMA NPs. (d) Normalized five-day real-time ECIS measurement of bEnd3brain epithelial cells, showing real-time data of attachment, proliferation, and response to fluorescent SMA NPs. Capacitance was recorded at 64 kHz. The dotted blue line indicates the time when the fluorescent SMA NPs were added to each well. (e) Capacitance values of bEnd3 cells on day five at 110 h in culture. (f) Cytotoxicity percentage of brain epithelial bEnd3 cells in response to contact with SMA NPs. In panels b, c, e, and f, the capacitances and cytotoxicities are compared to the negative control (brown bars). **** *p* < 0.0001. *n* = 4.

The ECIS assessment of cytotoxicity is consistent with the alamarBlue assay, indicating a common trend in both cell types in response to the addition of SMA NPs. Both measurements showed slight cytotoxicity in NIH 3T3 fibroblasts, although the ECIS measurements did not show statistically significant cytotoxicity. Furthermore, both assays showed very low cytotoxicity in bEnd3 endothelial cells. Regardless of the cell type or measurement method, there is no indication of dose-dependent cytotoxicity.

### 3.4 Barrier Function Condition Analysis

bEnd3 brain endothelial cells have been used for modeling the blood–brain barrier (BBB), since they present a similar permeability as the in vivo BBB.^30,31^ bEnd3 cells form strong barriers and can be affected by external agents or particles. Therefore, after analyzing the cytotoxicity impact of SMA NPs on NIH 3T3 fibroblasts and bEnd3 brain endothelial cells, bEnd3 cells were exposed to the NPs for an extended period to assess the effect on barrier function. In this experiment, 30000 cells were seeded in 200 μL of medium per well, with the number of replicates increased to five to improve confidence in our conclusions. Cells were cultured for 4 days until complete confluence was achieved, as confirmed by real-time measurements of resistance and capacitance (Figure 4). To study the barrier condition, a frequency of 4,000 Hz was used for resistance measurements. Capacitance at 64000 Hz was also used to monitor cell detachment. Once the bEnd3 cells reached confluence, barrier formation had commenced, and resistance values showed no significant differences between groups (Figure 4a). Subsequently, SMA NPs at the highest (146 μg/mL) and lowest (25 μg/mL) concentrations were added to each well. After 4 days of incubation with these NPs, cells exposed to the highest concentration exhibited significantly lower resistance compared to controls (two-way ANOVA, *p* < 0.01), indicating lower barrier strength; while cells treated with the lower concentration displayed only a slight, nonstatistically significant decrease compared to control (Figure 4b). These findings suggest that SMA NPs may slow barrier formation. However, the capacitance values decreased evenly for all groups (Figure 4c,d), indicating that cell death was not induced, regardless of the effect on barrier formation.

**Figure 4:**
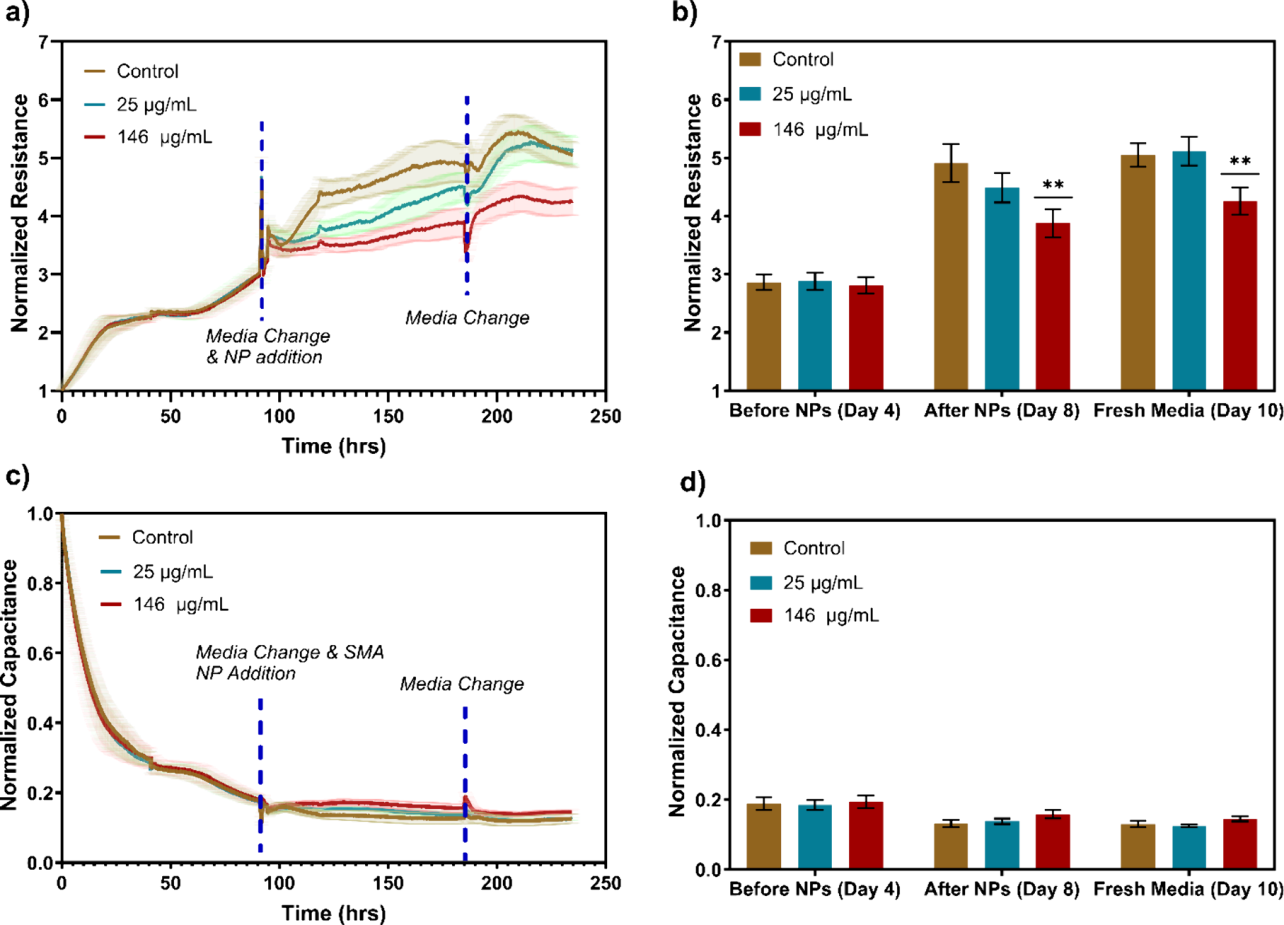
Barrier condition analysis of bENd3 brain epithelial cells after addition of SMA NPs at different concentrations. (a) Real-time ECIS resistance measurements at 4 kHz. (b) Comparison of resistance values between different experimental conditions, before adding SMA NPs on day 4, after SMA NPs addition on day 8, and after media replacement on day 10. (c) Real-time ECIS capacitance measurements at 64 kHz. (d) Comparison of normalized capacitance between different experimental conditions, before adding SMA NPs, after SMA NPs addition, and after media replacement. In panels b and d, the capacitance is compared to the negative control (brown bars). ** *p* < 0.0001. *n* = 5.

To determine if a medium change could enhance barrier formation, fresh medium without particles was introduced on day eight; 2 days later, wells with the lower NP concentration reached resistance levels similar to the controls, whereas wells with the highest concentration continued to show reduced resistance (Figure 4b,d; compare Fresh Media (Day 10) to After NPs (Day 8)). This persistence in altered barrier properties may be due to high-concentration SMA NPs remaining attached to the cells (Figures 5 and 6).

**Figure 5:**
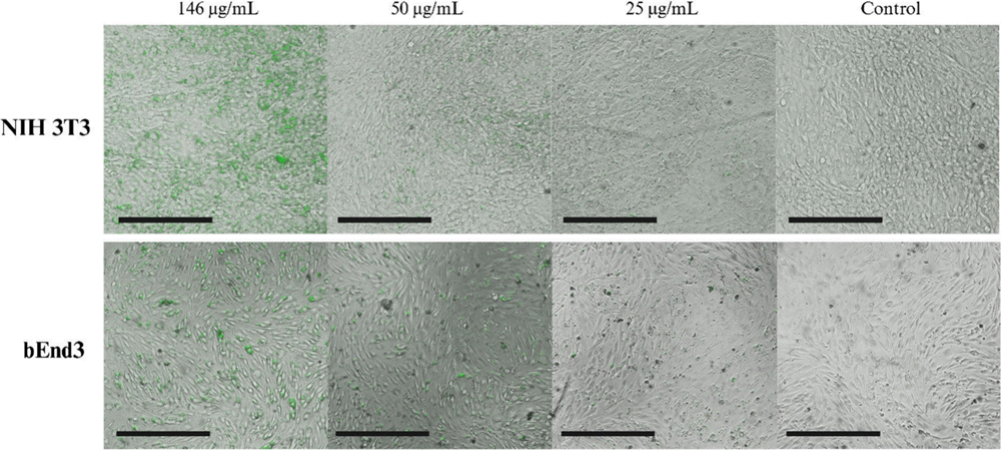
Fluorescence-phase contrast micrographs of randomly selected samples. Micrographs show NIH 3T3 fibroblasts (top panel) and bEnd3 brain endothelial cells (bottom panel) containing fluorescent SMA NPs (green) on day 7 at multiple concentrations. Scale bar = 275 μm.

**Figure 6:**
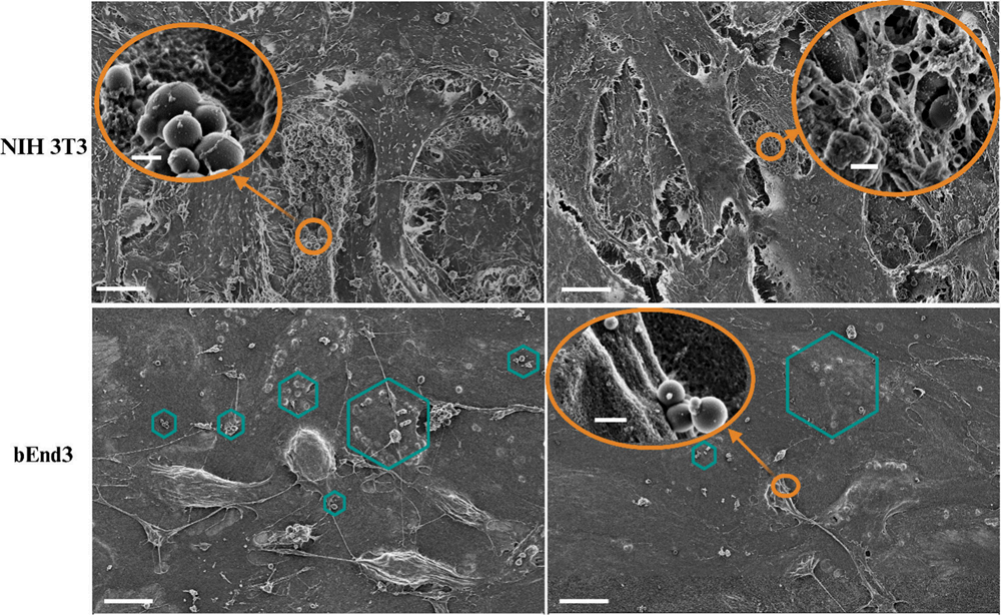
SEM images showing the cell–NP interaction after 7 days of culture with fluorescent SMA NPs. (Top panel) NIH 3T3 fibroblasts. (Bottom panel) bEnd3 brain endothelial cells. Turquoise hexagons point to SMA NPs attached to cells and intercalated into the extracellular matrix (ECM). The left and right panels are replicate samples. Scale bar = 10 μm. Zoomed micrograph scale bar = 400 nm.

The observed effects of SMA NPs on barrier function are noteworthy and consistent with previously reported interactions between NPs and endothelial barrier integrity. For example, carbon black NPs have been shown to cause endothelial barrier dysfunction via the RhoA/ROCK pathway, leading to cytoskeletal rearrangement and altered tight junctions.^32^ Similarly, zinc oxide NPs disrupted endothelial barriers by altering tight- and adherens-junction protein localization.^33^ Although our study did not examine the pathway of barrier function disruption, such mechanisms may contribute to the effects observed with SMA NPs.

### 3.5 Cell–SMA NPs Interaction Analysis

After 7 days of culture with SMA NPs and two media washes, both NIH 3T3 cells and bEnd3 cells were analyzed using phase contrast, fluorescence, and scanning electron microscopy. Noticeable clusters or patches of bright green fluorescence across the monolayer of both NIH 3T3 and brain epithelial cells were observed (Figure 5), which indicates the presence of fluorescent SMA NPs (green). As shown in Figure 1c, the SMA NPs display emission in the blue/green region (475–550 nm). The fibroblasts still appear well adherent, with no widespread cell detachment or abnormal morphology, suggesting no signs of cytotoxicity. The strong fluorescence indicates that the SMA NPs are on, within the cells and/or are distributed in the ECM. Similarly, bEnd3 brain endothelial cell coverage of the culture surface remains intact, with many cells showing typical elongation or cobblestone morphology. The SMA NP groups with the highest concentration (146 μg/mL) displayed higher levels of fluorescence, though lower concentration groups (50 and 25 μg/mL) still displayed some fluorescence.

Figure 5 shows a side-by-side comparison of various concentrations of fluorescent SMA NPs interacting with fibroblasts and endothelial cells after 7 days in culture. At all concentrations, cells exhibited confluent and healthy morphology, indicating that SMA NPs at concentrations ranging from 25 to 146 μg/mL did not overtly compromise cell adhesion or viability. The visual differences in fluorescence intensity across concentrations demonstrate the dose-dependent nature of NP distribution.

To further examine the cell-NP interaction, SEM was performed (Figure 6). These images confirm that both NIH 3T3 fibroblasts and brain epithelial bEnd3 cells maintain their characteristic morphology. The turquoise hexagons mark the SMA NPs distributed on the cell surfaces and intercalated into the ECM. The inclusion of zoomed micrographs, shown in orange, further unveils the fine structural details of individual SMA NPs and their close spatial interactions with cell membrane features. This detailed depiction underscores the capacity of SMA NPs to attach to and integrate with both the cellular surfaces and the extracellular milieu, offering valuable insights into the morphological and nanoscale interactions in a biologically active system.

## 4 Conclusions

In summary, we evaluated the cytotoxicity of oxygen-sensing SMA NPs to support their application in studying biomedical oxygen dynamics. SMA NPs were successfully synthesized, showing characteristic emission at 488 nm (green-yellow channel) and 650 nm (red channel). SMA NP biocompatibility was confirmed in NIH 3T3 fibroblasts and bEnd3 brain endothelial cells by performing real-time ECIS measurements along with the alamarBlue cell viability assay. At higher concentrations, SMA NPs affected barrier formation in bEnd3 cells. We also observed NP–cell interactions, including incorporation into the ECM, even after media changes and washing. Finally, we demonstrated that ECIS is an effective tool for evaluating the cytotoxicity of fluorescent materials. Although SMA NPs used in this study did not interfere with the alamarBlue readings, ECIS provides a useful alternative for assessing cytotoxicity in such cases and offers a valuable complementary approach.

Future work will elucidate the mechanisms of barrier function disruption and examine how the cell culture environment influences SMA NP chemistry and oxygen-sensing performance, offering deeper insight into NP–cell interactions. These factors are critical for the effective use of SMA NP sensors for monitoring in vitro oxygen distributions. Overall, our findings demonstrate SMA NP biocompatibility and their cellular interactions, underscoring the need for further mechanistic studies to support their application as in vitro oxygen sensors.

## Supplementary Material



## Data Availability

The data sets generated during the current study are available from the corresponding author upon request.

## Notes

Excitation spectra for Macrolex Yellow and PtTFPP (PDF)
