# Supplementary material for: Real-Time Monitoring of the Cytotoxic Effect of Oxygen-Sensitive Fluorescent Poly(styrene-maleic anhydride) Nanoparticles Using Electrical-Substrate Impedance Sensing
Source: ACS Appl Bio Mater. 2025 Oct 9;8(10):9322–31. doi: 10.1021/acsabm.5c01443 (PMC12541697; doi:10.1021/acsabm.5c01443)
Supplement: Supplementary file 1 [file mt5c01443_si_001.pdf]

Supplementary Information

Real-Time Monitoring of the Cytotoxic Effect of Oxygen-Sensitive Fluorescent Poly(styrene-maleic anhydride) Nanoparticles Using Electrical-Substrate Impedance Sensing

Fernando Pesantez Torres [a], Elijah C. Feret [a], Yubing Xie [a], and Susan T. Sharfstein [a,b,*]

[a] Department of Nanoscale Science and Engineering, College of Nanotechnology, Science and Engineering, University at Albany, State University of New York, 257 Fuller Road, Albany, NY 12203, USA

[b] The RNA Institute, University at Albany, State University of New York, 1400 Washington Ave., Albany, NY 12222

[*] Corresponding author
Susan T. Sharfstein, Ph.D., Professor, Department of Nanoscale Science and Engineering,
University at Albany, 257 Fuller Road, Albany, NY 12203, USA
Phone: 518-442-8642
Email: ssharfstein@albany.edu

Supplemental Figures S1 and S2

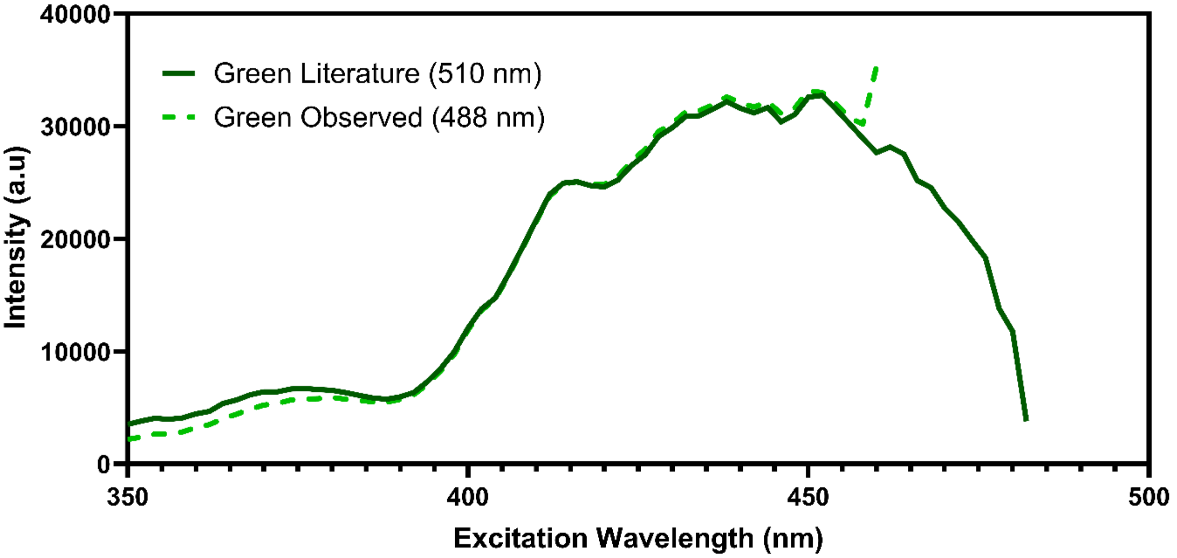

Figure S1. Excitation spectra for green emission of the reference dye. Excitation leading to emission in the green regime (reference dye) was assessed at emission wavelengths of 510 nm, corresponding to literature-reported fluorescence peaks, and 488 nm, corresponding to experimentally observed fluorescence peaks. Both samples were kept in ambient air. Peak excitation begins near 430 nm and continues until either spillover or until a sharp decrease beyond 460 nm.

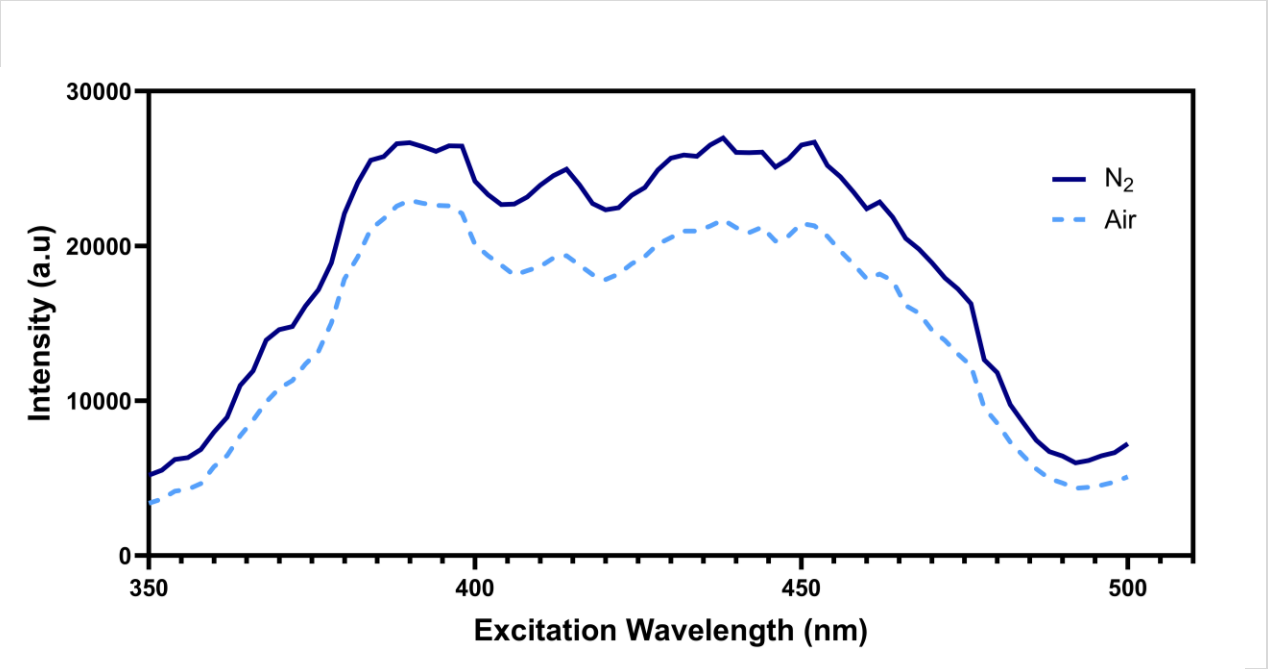

Figure S2. Excitation spectra for red emission of the oxygen sensor. Excitation leading to emission in the red regime (sensor dye) was assessed at 650 nm emission, which is the observed and literature-reported fluorescence peak. One sample was allowed to remain in the ambient air condition, while the other was bubbled with nitrogen prior to measurement. There are multiple excitation peaks, one near 390 nm and another broader peak near 440 nm. The sample exposed to nitrogen showed higher fluorescence intensity throughout.
